# The mediating effect of psychological empowerment on the relationship between work environment and clinical decision-making among midwives: a multicentre cross-sectional study

**DOI:** 10.1186/s12912-023-01282-0

**Published:** 2023-04-12

**Authors:** Jing Zeng, Sheng-Bin Guo, Qing-Xiang Zheng, Xiu-Wu Liu, Hui-Ming Lin, An-Fen Hu, Yan Yang, Bi-Rong Wei

**Affiliations:** 1 Fujian Maternity and Child Health Hospital, College of Clinical Medicine for Obstetrics & Gynecology and Pediatrics, Fujian Medical University, Fuzhou City, Fujian Province China; 2 Fujian Obstetrics and Gynecology Hospital, College of Clinical Medicine for Obstetrics & Gynecology and Pediatrics, Fujian Medical University, Fuzhou City, Fujian Province China; 3 The Affiliated Hospital of Guizhou Medical University, Guiyang City, Guizhou Province China; 4 The School of Nursing, Putian University, Putian City, Fujian Province China

**Keywords:** Midwife, Clinical decision-making, Work environment, Psychological empowerment, Structural equation model

## Abstract

**Background:**

Clinical decision-making is considered an essential behaviour in clinical practice. However, no research has been done to examine the associations among midwives’ clinical decision-making, work environment and psychological empowerment. Thus, this study aimed to determine the influence of work environment on midwives’ clinical decision-making and confirm the mediating role of psychological empowerment.

**Method:**

This study was designed as a multicentre cross-sectional study, and included 602 registered midwives from 25 public hospitals in China. A sociodemographic questionnaire, Work Environment Scale, Psychological Empowerment Scale and Clinical decision-making Scale were applied. A structural equation model was conducted to estimate the hypothesis model of the clinical decision-making among midwives and explore the potential mediating mechanism of midwives’ clinical decision-making. This model was employed maximum likelihood estimation method and bootstrapping to examine the statistical significance.

**Results:**

The mean score of clinical decision-making among midwives was 143.03 ± 14.22, at an intermediate level. The data of this hypothesis model fitted well, and the results showed that work environment positively affected psychological empowerment, which in turn positively affected clinical decision-making; psychological empowerment partly mediated the relationship between work environment and clinical decision-making among midwives.

**Conclusions:**

Midwives’ clinical decision-making could be promoted directly or indirectly by providing a healthy work environment and improving psychological empowerment. It is essential for hospital managers to pay attention to the assessment of the midwives’ work environment and actively improve it, such as establishing a supportive, fair and just workplace, and maintaining effective communication with midwives. Furthermore, managers can also promote midwives’ clinical decision-making behaviour by enhancing their psychological empowerment via enhancing job autonomy.

**Supplementary Information:**

The online version contains supplementary material available at 10.1186/s12912-023-01282-0.

## Introduction

Midwives play a vital role in reducing childbirth risks, providing low-cost and high-impact services for pregnant women and newborns [1]. Maternal and newborns health conditions are essential indicators of a country’s economic strength and social civility [2]. With sustainable development goals, maternal deaths should be less than 70 per 100,000 live childbirths by 2030 [3]. The world currently faces a shortage of 900,000 midwives, representing a third of the required global midwifery workforce. In addition, the COVID-19 crisis has exacerbated this problem [1]. The ratio of midwives to births in China is 1 in 4,000, far less than 6 midwives per 1,000 births as recommended by the World Health Organization [4]. The three-child policy which the government supports couples having a third child was recently implemented in China [5], which increases pressure and workloads on midwives’ clinical environments [5]. In the face of a severe shortage of midwives, managers must take measures to improve the efficiency of midwives while ensuring the quality of care.

Clinical decision-making is a complex process in which decision-makers combine theoretical knowledge and practical experience to make judgements about patients’ health problems [6]. It includes information handling, critical thinking, evaluating evidence, reflection and choosing the best practice [6]. Midwives with better clinical decision-making can quickly access the information from pregnant women, identify high-risk pregnancies, judge the progress of labour accurately, improve the science and effectiveness of interventions, and ensure the safety of pregnant women and newborns [7, 8]. Furthermore, effective clinical decision-making can improve midwives’ work efficiency and better cope with the current shortage of midwives [9, 10].

Evidence about the impacts of factors on clinical decision-making in midwifery is limited. However, there is evidence from other allied healthcare providers. For instance, previous studies have shown that factors such as clinical experience, education, patient situation, personnel resources, interpersonal relationships, organizational environment, feeling competent, and self-confidence are critical influences on the clinical decision-making process of nurses [11–13]. It is worth noting that there are some differences between nurses and midwives in terms of work conditions, service objects, and work modes. Furthermore, current research on the influencing factors of clinical decision-making primarily relies on qualitative research methods [14]. Therefore, a more comprehensive theoretical and practical model of the development of clinical decision-making among midwives, including related psychological factors, is needed to identify and implement relevant interventions.

The work environment generally refers to the physical, social, and psychological properties of the workplace that are perceived directly or indirectly by those who work there [15]. A positive work environment promotes nurses' clinical decision-making [12, 16], and further increase productivity due to the fact that a supportive work environment can improve work attitude and job satisfaction [17, 18]. A positive work environment concretely involves good cooperation between doctors and nurses, good nurse-patient relationships, support and care, and adequate staffing [15]. Therefore, understanding how midwives perceive the organizational environment is important to achieve an optimal work setting. However, little research has been done on the relationship between work environment and clinical decision-making among midwives. Given the importance of clinical decision-making among midwives [7, 8], it is necessary to focus on how clinical decision-making develops in midwives and interventions to improve their clinical decision-making ability. Thus, one purpose of our study is to explore the impact of the work environment on clinical decision-making among midwives.

According to social cognitive theory [19], individual behaviour is influenced by the external environment and self-perception. Given that clinical decision-making is a crucial behaviour in midwifery practice, we attempt to decipher the mechanism underlying this link from the perspective of self-perception. Psychological empowerment is a psychological variable that refers to an individual's self-perception of their job, including its meaning, self-efficacy, self-determination, and influence [20]. According to job demand-resource model, as a crucial job resource, psychological empowerment influences nurses' attitudes and behaviours [21]. For instance, autonomy in nursing practice enables effective clinical decision-making [22], and feeling competent and self-confident are crucial factors in this process [12]. Thus, we hypothesize that psychological empowerment can enhance clinical decision-making among midwives.

Meanwhile, psychological empowerment can influence work behaviour by generating self-evaluation of work, which is based on the individual's subjective assessment of the work environment [23]. A supportive work environment can enhance nurses' perceptions of their abilities and self-efficacy, which gives them the confidence to meet job requirements and appreciate the intrinsic value of their tasks [24]. Improving the work environment is conducive to higher levels of psychological empowerment [25]. Therefore, we propose that psychological empowerment may mediate the relationship between the work environment and clinical decision-making behaviour among midwives.

**Fig. 1:**
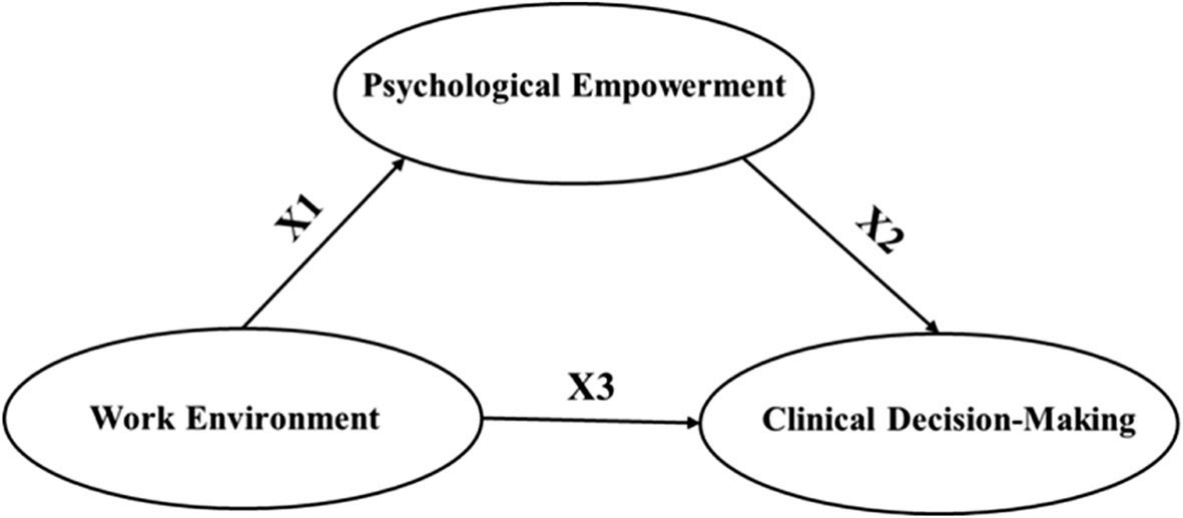
Theoretical model and hypotheses

Base on above literature review, this survey aimed to assess the clinical decision-making among midwives and explore the potential mediating mechanism of midwives’ clinical decision-making. Three hypotheses in this study are presented in Fig. 1.

Hypothesis 1: Work environment is positively related to clinical decision-making among midwives.

Hypothesis 2: Work environment positively affects psychological empowerment, and psychological empowerment positively affects clinical decision-making among midwives.

Hypothesis 3: Work environment could positively predict clinical decision-making and partially affect it via psychological empowerment.

## Methods

### Study design and participants

A multicentre cross-sectional design study was carried out in 25 Chinese public hospitals from July 2021 to August 2021. This study was conducted according to the guideline of Strengthening the Reporting of Observational Studies in Epidemiology (Additional file 1). The sample size was calculated by PASS 15. According to Li’s study [26], the standard deviation of the clinical decision-making score among 583 midwives was 11.22. Given this criterion (Confidence Interval = 0.95, Confidence interval precision = 10% of the standard deviation), 385 midwives were needed. After a non-response rate of 20% was considered, the minimum sample size was 462 midwives.

602 midwives were selected through convenient sampling from 25 hospitals. The inclusion criteria of this study were as follows: (a) registered midwives; (b) midwives who worked in the obstetric ward, delivery room, and obstetrics clinic; (c) midwives who volunteered to participate in the study. The exclusion criteria included: (a) midwives in turn or probation period; (b) midwives gone to or came from other hospitals for advanced training; (c) midwives on maternity or sick leave; (d) midwives who had less than three months of experience.

### Measures

The researchers designed the sociodemographic questionnaire, including midwives’ age, years of work experience, marital status, fertility status, education level, monthly income, hospital rank, professional rank, professional position, employment reasons, and professional identity.

#### Nursing Work Environment Scale (NWES)

This questionnaire developed by Shao [27] et al., and was used widely to measure the work conditions of nurses or midwives in mainland China. The Chinese version has a good reliability with Cronbach’s alpha value ranging from 0.799 to 0.946 [27]. It contained seven dimensions and 26 items: professional development, support and care, recognition of value, clinical autonomy, salary and welfare, staffing adequacy, and nurse-physician relations. It was scored on a 6-point Likert scale, and the total score of the questionnaire was 26 ~ 156 scores. The higher the score, the better the work environment. In the current study, Cronbach’s alpha value was 0.963.

#### Psychological Empowerment Scale (PES)

This questionnaire was developed by Spreitzer [28] and translated as the Chinese version by Li et al. [29], and was used widely to measure the psychological empowerment of employee in mainland China. It including four subscales with a total of 12 items: meaning, self-efficacy or competence, self-determination, and impact. It was scored on a 5-point Likert scale, and the total score was determined to be the total of each item. The higher the scores, the greater the heightened sense of psychological empowerment. The Chinese version of the PES was used widely and had good reliability with Cronbach’s alpha value ranging from 0.72 to 0.86 [29]. In this study, Cronbach’s alpha value for this questionnaire was 0.939.

#### Clinical Decision-Making in Nursing Scale (CDMNS)

This questionnaire was developed by Jenkins & Helen [30] and translated as a Chinese version by Guo [31], and was used widely to measure the Clinical Decision-Making ability of nurses or midwives in mainland China. It had good reliability with Cronbach’s alpha value was 0.72, and including four subscales with a total of 40 items: search for alternatives or options, canvassing of objectives and values, evaluation and re-evaluation of consequences, and search for information. This questionnaire was scored on a 5-point Likert scale ranging from 40 to 200 and the total score divided into three levels: 40.00 ~ 93.33 points for low ability, 93.34 ~ 146.67 points for intermediate ability, and 146.68 ~ 200.00 points for high ability [31]. The higher the scores, the better the make decision-making. In the current study, Cronbach’s alpha coefficient of this questionnaire was 0.860.

### Data collection

The questionnaires in the study were collected online through Wen Juan Xing and WeChat. Before the survey, consent was obtained from the 25 hospitals. Then, the head midwives of these hospitals were trained and informed by the researchers regarding the research purpose, the requirements of choosing participants, and the instructions on filling out questionnaires through the online meeting. Respondent anonymity and data confidentiality were guaranteed. Next, the researchers created this survey’s quick response (QR) code through Wen Juan Xing, which was the most prominent professional and free online survey platform in China [32]. And then, the questionnaires’ QR code was sent by the researcher to the head midwives through WeChat, an online communication platform [33]. After that, the head midwives sent the questionnaires’ QR codes to eligible participants through WeChat in the morning meeting. Participants could sweep the questionnaires’ QR codes in WeChat and complete the questionnaire. The Wen Juan Xing platform allows only one submission for a single WeChat account. The participants could not submit their questionnaires if data were incomplete or missing.

### Data analysis

Data were analysed by using SPSS version 26.0 and AMOS version 26.0. Enumeration data were described in percentage (%), the measurement data that met normal distribution were described with mean and standard deviation (SD), and the measurement data that satisfied non-normal distribution were presented using the median with interquartile range. Spearman or Pearson correlation analyses were conducted to analyse the scores of CDMNS, PES, and NWES. Stepwise multiple linear regression was utilized to identify factors influencing the clinical decision-making among midwives. All independent variables were entered into the multivariable regression models. The variance inflation factor (VIF) was inspected for evidence of multicollinearity in the model. Data were considered statistically significant if *p* < 0.05.

AMOS was used for structural equation modelling (SEM). The SEM employed maximum likelihood estimation method, and the model’s goodness-of-fit indices were evaluated through relative and absolute indices, which included the comparative fit index (CFI), the Tucker–Lewis’s index (TLI), incremental fit index (IFI), normed fit index (NFI), goodness-of-fit index (GFI), adjusted goodness-off-index (AGFI), root mean square error of approximation (RMSEA) and chi-square/degrees of freedom ratio (χ^2^/df). A hypothetical model that met the following threshold values was considered adequate: CFI > 0.90, TLI > 0.90, IFI > 0.90, NFI > 0.90, GFI > 0.80, AGFI > 0.80 and RMSEA < 0.08 and χ^2^/df < 3.00 [34]. Bootstrapping was performed to examine the statistical significance of the indirect and total effects of the model.

### Ethical approval

The study was approved by the Ethical Committee of Fujian Maternity and Child Health Hospital, College of Clinical Medicine for Obstetrics & Gynecology and Pediatrics, Fujian Medical University, Fuzhou City, China (No:2021YJ060). 

## Result

### Participant characteristics

Registered midwives accomplished 602 questionnaires through convenient sampling from 25 hospitals. Among them, 520 were valid (86.37%). The reason for the high non-response rate was that in order to analyse the data accurately, some data, such as inconsistent answers, records with a straight line, and a response time of less than 5 min were excluded in the final analysis (Additional file 2). The midwives’ mean age and job tenures were 34.86 years (SD = 7.64) and 13.54 years (SD = 8.45), respectively. Most participants had a bachelor degree (51%). Their monthly income ranged from 5001 to 8000RMB (1RMB = 0.1446USD), and 80% of the women were married. The work departments of the participants mainly included the obstetric ward, delivery room, and obstetrics clinic (50%, 46%, and 4%, respectively) from tertiary (61%) and secondary (39%) hospitals. Approximately 45% of the participants had a junior professional rank, and 68% were clinical midwives. Other demographic data are presented in Table 1.

**Table 1:** General characteristics of subjects (*N* = 520)

Variables	Categories	*N* (%)
Age(years)	≤ 25	41(7.9)
	26–30	133(25.6)
	31–35	133(25.6)
	36–40	103(19.8)
	41–45	55(10.6)
	≥ 46	55(10.6)
Job tenure(years)	≤ 5	93(17.9)
	6–10	151(29.0)
	11–15	86(16.5)
	16–20	84(16.2)
	21–25	48(9.2)
	≥ 26	58(11.2)
Education level	Technical secondary school degree	16(3.1)
	Junior college degree	234(45.0)
	Bachelor degree	267(51.3)
	Master degree or above	3(0.6)
Marital status	Single	94(18.1)
	Married	415(79.8)
	Divorced	9(1.7)
	Widowed	2(0.4)
Fertility status	Childless	127(24.4)
	One child	206(39.6)
	Two children	185(35.6)
	Three children or above	2(0.4)
Hospital rank	Secondary	200(38.5)
	Tertiary	320(61.5)
Hospital location	Fuzhou City	156(30.0)
	Xiamen City	84(16.2)
	Putian City	54(10.4)
	Quanzhou City	63(12.1)
	Zhangzhou City,	47(9.0)
	Longyan City	42(8.1)
	Nanping City	36(6.9)
	Sanming City	20(3.8)
	Ningde City	18(3.5)
Work department	Obstetric ward	263(50.6)
	Delivery room	238(45.8)
	Obstetrics Clinic	19(3.7)
Professional rank	None	68(13.1)
	Junior	235(45.2)
	Intermediate	159(30.6)
	Senior	58(11.2)
Job category	Temporarily employed midwives	204(39.2)
	Permanently employed midwives	314(60.4)
	Other	2(0.4)
Monthly income (yuan)	≤ 5000	123(23.7)
	5001–8000	260(50.0)
	8001–10,000	71(13.7)
	> 10,000	66(12.7)
Work objective	Love nursing	207(39.8)
	Satisfy parents’ expectation	18(3.5)
	Survival need	199(38.3)
	Other	96(18.5)
Case discussion	No	47(9.0)
	1 time per week	35(6.7)
	1 time per month	310(59.6)
	1 time per 3 months	81(15.6)
	1 time per 6 months	47(9.0)
Whether to participate in academic activities related to clinical decision-making	No	228(43.8)
	Academic Activities in the Hospital	231(44.4)
	Academic activities in the province	43(8.3)
	National Academic Activities	18(3.5)
Professional identity	Strongly agree	293(56.3)
	Agree	143(27.5)
	Neutrality	83(16.0)
	Disagree	0(0)
	Strongly disagree	1(0.2)

*Abbreviations*: *SD* standard deviation

### Analyses of multicollinearity

The result of analyses of multicollinearity showed that all predictive variables’ variance inflation factors were less than two. Therefore, there was no severe problem of multicollinearity in this study.

### Common-method bias

All data from NWES, PES, and CDMNS were tested using Harman’s one factor-test for common-method bias. The unrotated exploratory factor analysis results extracted 14 factors with characteristic roots greater than one. The maximum factor variance explained was 29.413% (less than 40%). Thus, there was no common-method severe bias in this study.

### Comparison of the NWES, PES and CDMNS scores

The median score of the NWES was 4.81, ranging from 4.19 points to 5.15 points. The highest score was found in value recognition [5.00 (4.67, 5.33)], followed by midwives-physician relations [5.00 (4.50, 5.25)] and clinical autonomy [5.00 (4.50, 5.25)]. The median score of the PES was 3.96, ranging from 3.58 points to 4.42 points, and meaning had the highest score [4.00 (4.00, 5.00)]. In addition, the median score of CDMNS was 3.58 points, ranging from 3.33 points to 3.83 points. The highest score was found in Evaluation and re-evaluation of consequences [3.80 (3.40,4.10)], followed by Canvassing of objectives and values [3.60 (3.30,3.80)], Search for alternatives or options [3.50 (3.23,3.90)] and Search for information [3.40 (3.10,3.70)]. The results are presented in Table 2.

**Table 2:** The comparison of NWES, PES, and CDMNS Score (*N* = 520)

Variables	Mean ± SD	median (IQR)
NWES (total score: 26–156)	122.39 ± 19.61	125.00(109.00,134.00)
NWES (potential point: 1–6)	4.71 ± 0.75	4.81 (4.19,5.15)
Professional development	4.63 ± 0.94	4.80 (4.00,5.20)
Support and care	4.67 ± 0.96	5.00 (4.00,5.25)
midwives–physician relations	4.84 ± 0.80	5.00 (4.50,5.25)
Recognition of value	5.05 ± 0.67	5.00 (4.67,5.33)
Clinical autonomy	4.90 ± 0.72	5.00 (4.50,5.25)
Salary and welfare	4.12 ± 1.29	4.33 (3.08,5.00)
Staffing adequacy	4.71 ± 0.88	5.00 (4.33,5.00)
PES (total score: 12–60)	47.45 ± 6.97	43.00(47.50,53.00)
PES (potential point: 1–5)	3.95 ± 0.58	3.96 (3.58,4.42)
Meaning	4.17 ± 0.68	4.00 (4.00,5.00)
Self-determination	4.06 ± 0.68	4.00 (3.67,4.67)
Self-efficacy or competence	4.19 ± 0.57	4.00 (4.00,4.67)
Impact	3.40 ± 0.88	3.33 (3.00,4.00)
CDMNS (total score: 40–200)	143.03 ± 14.22	133.00(143.00,153.00)
CDMNS (potential point: 1–5)	3.58 ± 0.36	3.58 (3.33,3.83)
Search for alternatives or options	3.57 ± 0.43	3.50 (3.23,3.90)
Canvassing of objectives and values	3.57 ± 0.37	3.60 (3.30,3.80)
Evaluation and re-evaluation of consequences	3.76 ± 0.52	3.80 (3.40,4.10)
Search for information	3.40 ± 0.37	3.40 (3.10,3.70)

*Abbreviation*: (1) NWES: the scores of Nursing Work Environment scale; (2) PES: the score of the Psychological Empowerment Scale; (3) CDMNS: the score of Decision Making in Nursing Scale

### Correlation analysis of the NWES, PES and CDMNS scores

The results of the correlation analysis of the NWES, PES, and CDMNS scores are shown in Table 3. The NWES score and its factors were positively correlated not only with the PES score and its factors (*p* < 0.01) but also with the CDMNS score and its factors (*p* < 0.01). The PES score and its factors were positively correlated with the CDMNS score and its factors (*p* < 0.01).

**Table 3:** Spearman correlation coefficients of NWES, PES and CDMNS Score (R, *N* = 520)

Variables	NWES	NWES1	NWES2	NWES3	NWES4	NWES5	NWES6	NWES7	PES	PES1	PES2	PES3	PES4	CDMNS	CDMNS1	CDMNS2	CDMNS3	CDMNS4
NWES	1.000																	
NWES1	0.920^a^	1.000																
NWES2	0.893^a^	0.834^a^	1.000															
NWES3	0.828^a^	0.709^a^	0.703^a^	1.000														
NWES4	0.781^a^	0.650^a^	0.665^a^	0.753^a^	1.000													
NWES5	0.763^a^	0.660^a^	0.664^a^	0.629^a^	0.662^a^	1.000												
NWES6	0.848^a^	0.747^a^	0.689^a^	0.610^a^	0.599^a^	0.555^a^	1.000											
NWES7	0.838^a^	0.731^a^	0.721^a^	0.706^a^	0.660^a^	0.589^a^	0.685^a^	1.000										
PES	0.695^a^	0.601^a^	0.600^a^	0.618^a^	0.678^a^	0.658^a^	0.582^a^	0.546^a^	1.000									
PES1	0.642^a^	0.566^a^	0.542^a^	0.544^a^	0.648^a^	0.536^a^	0.553^a^	0.534^a^	0.825^a^	1.000								
PES2	0.625^a^	0.528^a^	0.563^a^	0.559^a^	0.611^a^	0.580^a^	0.510^a^	0.511^a^	0.883^a^	0.717^a^	1.000							
PES3	0.517^a^	0.404^a^	0.450^a^	0.509^a^	0.608^a^	0.521^a^	0.413^a^	0.449^a^	0.785^a^	0.621^a^	0.707^a^	1.000						
PES4	0.499^a^	0.459^a^	0.419^a^	0.440^a^	0.413^a^	0.511^a^	0.437^a^	0.365^a^	0.778^a^	0.452^a^	0.559^a^	0.433^a^	1.000					
CDMNS	0.453^a^	0.365^a^	0.383^a^	0.431^a^	0.489^a^	0.435^a^	0.378^a^	0.365^a^	0.445^a^	0.446^a^	0.391^a^	0.475^a^	0.241^a^	1.000				
CDMNS1	0.392^a^	0.320^a^	0.334^a^	0.381^a^	0.417^a^	0.395^a^	0.323^a^	0.318^a^	0.428^a^	0.409^a^	0.376^a^	0.424^a^	0.278^a^	0.879^a^	1.000			
CDMNS2	0.458^a^	0.374^a^	0.388^a^	0.417^a^	0.467^a^	0.417^a^	0.384^a^	0.403^a^	0.440^a^	0.416^a^	0.383^a^	0.443^a^	0.276^a^	0.773^a^	0.605^a^	1.000		
CDMNS3	0.374^a^	0.304^a^	0.314^a^	0.361^a^	0.412^a^	0.366^a^	0.323^a^	0.285^a^	0.361^a^	0.363^a^	0.314^a^	0.409^a^	0.180^a^	0.893^a^	0.709^a^	0.603^a^	1.000	
CDMNS4	0.311^a^	0.248^a^	0.264^a^	0.303^a^	0.353^a^	0.297^a^	0.238^a^	0.245^a^	0.279^a^	0.325^a^	0.264^a^	0.337^a^	0.078	0.768^a^	0.607^a^	0.452^a^	0.582^a^	1.000

*Abbreviation*: (1) NWES: the scores of Nursing Work Environment scale; NWES 1–7: factor score of Nursing Work Environment scale representing "professional development," "recognition of value," "support and care," "Nurse–physician relations," "support and care," "clinical autonomy," "salary and welfare," and "staffing adequacy" respectively. (2) PES: the score of the Psychological Empowerment Scale; C-PES 1–4: factor score of Psychological Empowerment Scale, representing "meaning," "self-efficacy or competence," "self-determination" and "impact," "respectively." (3) CDMNS: the score of Decision Making in Nursing Scale, C-CDMNS1-4: factor score of Decision Making in Nursing Scale, representing "search for alternatives and options scale," "canvassing of objectives" and "values scale," "evaluation and re-evaluation of consequences scale," and "Search for information," respectively. ^a^correlation is significant at the 0.01 level

### Factors influencing clinical decision-making among midwives

Collinearity diagnosis shows that there was no multicollinearity for all independent variables. All variables were entered in the stepwise multiple linear regression model for analysis. After adjusted analysis, significant factors influencing clinical decision-making included work environment, psychological empowerment, professional rank, professional identity, fertility status and whether to participate in academic activities related to clinical decision-making in the final regression model, which explained 27.4% of the total variance of clinical decision-making (F = 33.496, *p* < 0.001). The result is shown in Table 4.

**Table 4:** Multiple linear regression analysis for the factors of clinical decision-making (*N* = 520)

	Beta	SE	*t*	*p*	*B*
Constant	88.262	4.431	19.919	0.000	
work environment	0.150	0.040	3.742	0.000	0.206
psychological empowerment	0.462	0.111	4.177	0.000	0.226
Professional rank	1.771	0.688	2.575	0.010	0.106
Whether to participate in academic activities related to clinical decision-making	2.374	0.750	3.165	0.002	0.127
Professional identity	2.155	0.804	2.680	0.008	0.114
Fertility status	-1.588	0.730	-2.177	0.030	-0.087

*R*^*2*^ = 0.283, Adjusted *R*^*2*^ = 0.274, F = 33.496

*Abbreviations*: *SE* standard error, *B* standardized beta

### Fitness of the hypothetical path model

**Fig. 2:**
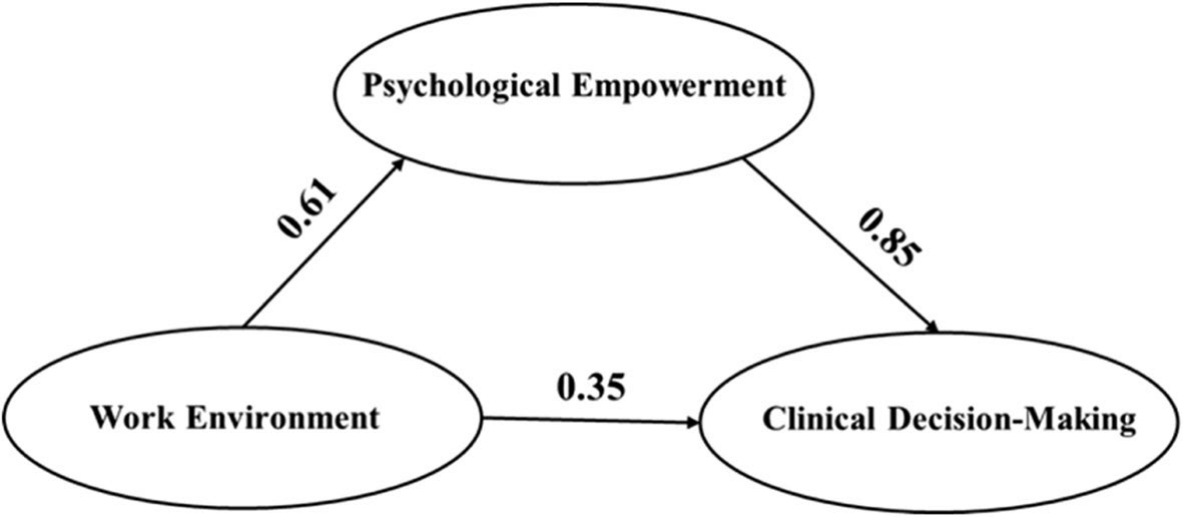
Model of work environment for midwives and psychological empowerment on clinical decision-making

The model of the work environment and psychological empowerment amongst midwives and their effect on clinical decision-making is shown in Fig. 2. The NWES score was considered an independent variable, and the CDMNS score was set as a dependent variable. The PES score was considered an intermediary variable for constructing a structural equation model and testing its hypothesis relation.

Before building a hypothetical model, the researcher conducted validity analyses on NWES, PES, and CDMNS. According to the structural validity results “professional development” and “recognition of value,” subscales of NWES were highly correlated. It may be related to the fact that professional development and recognition of value are essential components of a sense of decent work [35]. The concept of “decent work” refers to individuals’ right to have free, equal and safe access to decent work opportunities in conditions of human dignity [35]. In addition, a subsequent correlation analysis of NWES also confirmed this conclusion. Therefore, “professional development” and “recognition of value” were removed from the final mediation model. The factor loadings between the latent variables and the respective observed variables in this model were 0.62 ~ 0.87. The path coefficients of the work environment and clinical decision-making, the work environment and psychological empowerment and psychological empowerment and clinical decision-making were 0.35, 0.61 and 0.85, respectively. All path coefficients in the model were statistically significant (*p* < 0.05). Furthermore, the hypothetical model presented acceptable fits (CFI = 0.970, TFI = 0.961, IFI = 0.970, NFI = 0.956, GFI = 0.951, AGFI = 0.924, RMSEA = 0.062 and χ^2^/*df* = 2.984).

The specific effect values of each path in this model are shown in Table 5. Hypothesis 1 was supported, i.e., work environment had a positive total and direct effect on clinical decision-making [β = 0.865, *p* < 0.01, 95% confidence interval (95% CI; 0.688, 1.503); β = 0.352, *p* < 0.1, 95% CI (0.03, 0.663), respectively]. Hypothesis 2 was also supported; in other words, psychological empowerment had a positive total and direct effect on clinical decision-making [β = 0.846, *p* < 0.01, 95% CI (0.448, 1.275)]. In addition, the work environment had a positive direct effect on psychological empowerment [β = 0.606, *p* < 0.01, 95% CI (0.534, 0.684)], and the work environment indirectly affected clinical decision-making [β = 0.513, *p* < 0.01, 95% CI (0.278, 0.782)] via psychological empowerment, thereby supporting Hypothesis 3. Therefore, the model fitted the data well. The results also indicated that the work environment and psychological empowerment positively affected clinical decision-making. We also found that psychological empowerment mediated between work environment and clinical decision-making among midwives.

**Table 5:** The total effects, direct effects, and indirect effects of every path in this model

		Blas-corrected 95%CI^a^		
**Estimate**	**β**	**Lower**	**Upper**	***P***
**Total effects**				
Work Environment → Clinical Decision-Making	0.865	0.688	1.053	< 0.01
Psychological Empowerment → Clinical Decision-Making	0.846	0.448	1.275	< 0.01
**Direct effects**				
Work Environment → Psychological Empowerment	0.606	0.534	0.684	< 0.01
Psychological Empowerment → Clinical Decision-Making	0.846	0.448	1.275	< 0.01
Work Environment →Clinical Decision-Making	0.352	0.03	0.663	< 0.1
**Indirect effects**				
Work Environment → Psychological Empowerment →Clinical Decision-Making	0.513	0.278	0.782	< 0.01

^a^Means that 95% bias-corrected bootstrap confidence interval

## Discussion

This study described the current status of clinical decision-making among midwives, and explored the associations of work environment, psychological empowerment and clinical decision-making as well as confirmed the mediating role of psychological empowerment in this relationship among Chinese midwives. There were three significant findings. First, midwives’ clinical decision-making were at intermediate level. Second, work environment and psychological empowerment were positively associated with clinical decision-making. Third, the work environment could positively predict clinical decision-making and partially affect it via psychological empowerment.

### Status of clinical decision-making in midwives

The birth outcome was directly influenced by midwives’ clinical decision-making [36]. We found that the CDMNS score of midwives was 143.03 (SD = 14.22), which indicated that clinical decision-making ability among midwives was at an intermediate level and similar to the results from Li et al. [26]. However, this score was lower than Saudi Arabian nurses [37]. It must be related to the fact that Saudi Arabian nurses had received basic courses and additional courses which improve nurses’ critical thinking skills and clinical decision-making skills [37]. In addition, the prevalence of exam-oriented education in China at the end of the twentieth century also seems to explain this phenomenon. It is an educational system that focuses on test-taking skills, memorisation and problem solving, leading to students’ emphasis on theory over practice [38].

Our results also indicated that searching for information, the lowest scores among clinical decision-making subscales, was the most urgent competency for midwives, consistent with Chinese junior nurses [16]. It may be related to midwives’ lack of knowledge about ways to search for information. Plus, the heavy daily workload prevents midwives from developing systematic and scientific study habits in their off-duty time, which could also explain this phenomenon. Thus, the managers should provide courses about searching for e-books, up-to-date literature and guides, which can help midwives explore knowledge quickly and effectively. They ought to encourage midwives to develop lifelong self-directed learning habits, which can help midwives update their professional knowledge and working skills so as to provide better services for pregnant women and newborns [39].

### Work environment and psychological empowerment were positively associated with clinical decision-making

The results indicated that the work environment positively influenced the clinical decision-making of midwives; consistent with previous studies showed that work environment factors, such as nursing management, nurse-physician relationships, nurse-patient relationship and workload are considered predictors of clinical decision-making [14, 40]. According to the principle of reciprocity in social exchange theory [41], when employees feel favourable treatment from the organization and managers, they will give back to the organization and their managers with a serious attitude and hard work behaviour. Providing sufficient resources and an excellent work atmosphere is an investment by hospitals in midwives. When midwives perceive a healthy work environment, they easily produce an organizational identity psychologically and participate actively in decision-making to provide better care for pregnant women. Therefore, it is essential for hospital managers to pay attention to the assessment of the midwives’ work environment and actively improve it.

In addition, we found that the work environment positively influenced psychological empowerment for midwives. In line with prior studies, a supportive work environment likely improves nurses’ psychological empowerment and the supportive work in which care and trust prevail [42–44]. Moreover, employees’ relationships tend to be people-oriented and based on sharing. The feeling of psychological empowerment increases as leader approachability, group effectiveness and group value increase [45]. Consistent with previous findings, our results indicated that psychological empowerment positively affected clinical decision-making among midwives. Psychological empowerment is closely linked to nurses’ clinical decision-making [46], and as an intrinsic motivator, it can positively influence behaviour by changing internal beliefs [47]. Therefore, managers can create a healthy work environment for midwives and value their psychological empowerment to promote positive work outcomes.

### Work environment positively predicted clinical decision-making and can affect it partially through psychological empowerment

These findings suggest that the work environment had a direct positive effect on midwives’ clinical decision-making and could have an indirect positive influence through psychological empowerment. Many researchers validated psychological empowerment as a mediator of work engagement, quality of care, career satisfaction and propensity to leave [42, 48, 49]. However, few studies have explored the role of psychological empowerment in the relationship between the work environment and clinical decision-making. An individual’s internal motivation can be effectively expressed in terms of the positive stimulus from the external environment [50]. Combined with our research, we considered a healthy work environment to be a positive external stimulus that provided midwives with transformational leadership, support from their organisation, midwives-physician cooperation, good interpersonal relationships and adequate midwife staffing. Midwives’ intrinsic motivation can be effectively expressed in this supportive work environment, thus stimulating a sense of self-efficacy and contributing to midwives’ clinical decision-making behaviour [12]. These findings indicated that improving the work environment can directly enhance clinical decision-making and also enhance clinical decision-making through improving midwives' psychological empowerment. Future research should focus on the impact of psychological empowerment related interventions on clinical decision-making behaviour in midwives.

### Limitations of this study

This study had several limitations that need to be addressed in future research. Firstly, the sample size of the study was limited to only 602 midwives in Fujian Province. To enhance the generalizability of the results, future studies should cover a wider range of provinces and increase the sample size. Secondly, the study was conducted in 25 public hospitals, and it is possible that there were systematic differences in clinical decision-making between midwives working in public hospitals and those in other healthcare institutions. Therefore, future research could compare the differences in midwives' clinical decision-making in hospitals of different natures. Thirdly, we acknowledge that there are other individual or organisational factors influencing clinical decision-making, which may have moderating or mediating effects. Therefore, including and exploring more variables in future research is needed. Fourthly, the study's cross-sectional design limits the ability to draw causal conclusions, hence further research using a longitudinal approach is needed to establish the direction of the relationship.

## Conclusion

Midwives' clinical decision-making is at an intermediate level, with a particular need for improvement in information searching. Additionally, the work environment has a positive effect on psychological empowerment, which in turn positively influences clinical decision-making. Psychological empowerment also mediates the relationship between the work environment and clinical decision-making among midwives. Therefore, it is essential for hospital managers to actively improve midwives' work environment by establishing a supportive, fair and just workplace, maintaining effective communication with midwives, assisting midwives with career development and planning, and demonstrating care for their well-being. Furthermore, managers can promote midwives' clinical decision-making behaviour by enhancing their psychological empowerment via enhancing job autonomy and improving work impact.

## Supplementary Information

**Additional file 1.** STROBE Statement—EQUATOR checklist of items that should be included in reports of observational studies.

**Additional file 2.** Details of the deleting process of the data.

## Data Availability

The datasets used and/or analysed during the current study available from the corresponding author on reasonable request.

## Abbreviations

PES: Psychological Empowerment Scale
NWES: Nursing Work Environment Scale
CDMNS: Clinical Decision-Making in Nursing Scale
QR: Quick response
CFI: Comparative Fit Index
TLI: Tucker-Lewis’s Index
IFI: Incremental Fit Index
NFI: Normed fit Index
GFI: Goodness-of-Fit Index
AGFI: Adjusted Goodness-off-Index
RMSEA: Root Mean Square Error of Approximation
(χ2/df): Chi-square/Degrees of Freedom ratio
