# Supplementary material for: The mediating effect of psychological empowerment on the relationship between work environment and clinical decision-making among midwives: a multicentre cross-sectional study
Source: BMC Nurs. 2023 Apr 12;22:116. doi: 10.1186/s12912-023-01282-0 (PMC10092914; doi:10.1186/s12912-023-01282-0)

**Additional file 2**

**Details of the deleting process of the data**

Two members of the research team worked together to screen out the undesirable records. The records which belonged to the following criteria were excluded:

1) response time less than 5 minutes (21 records);

2) answers were straight-line or inconsistent (61 records);

In total, we deleted 82 records as we believe those records might have the response bias.


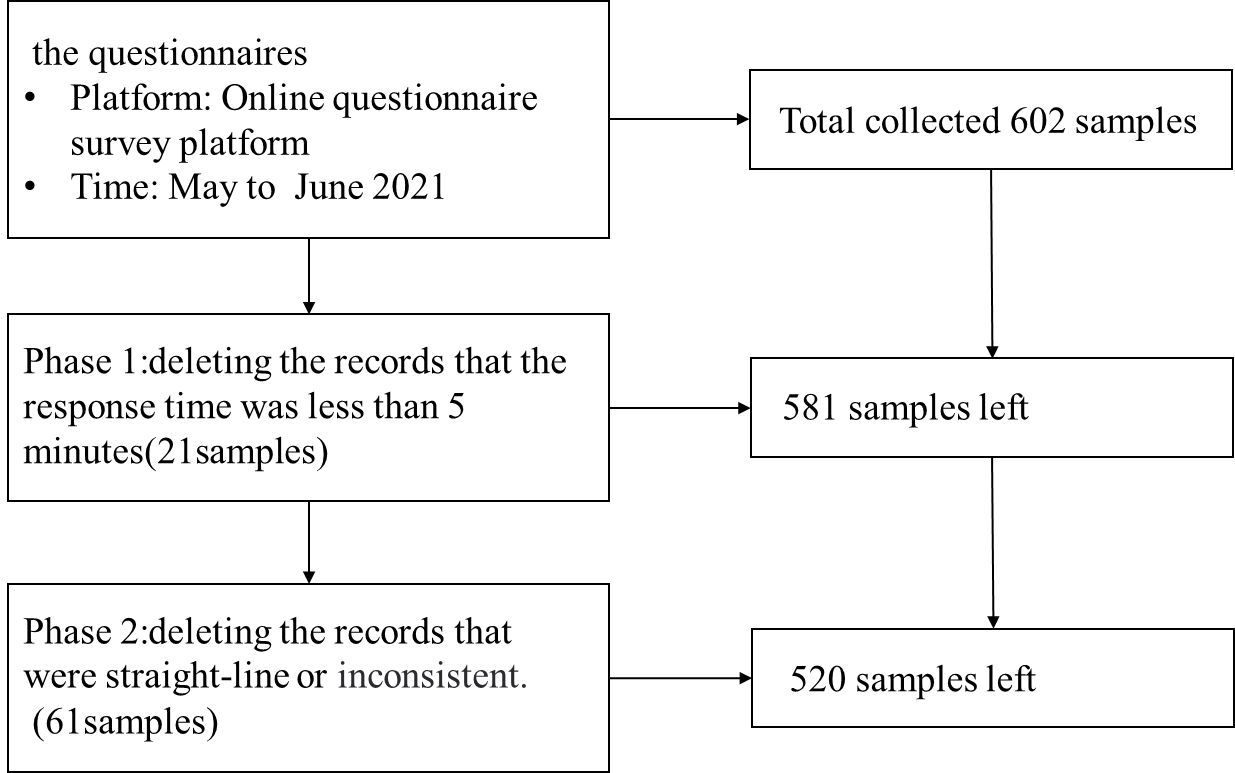

Supplement: Supplementary file 2 — Additional file 2. Details of the deleting process of the data. [file 12912_2023_1282_MOESM2_ESM.docx]
